# Reduced HRAS^G12V^-Driven Tumorigenesis of Cell Lines Expressing KRAS^C118S^

**DOI:** 10.1371/journal.pone.0123918

**Published:** 2015-04-22

**Authors:** Lu Huang, Christopher M. Counter

**Affiliations:** 1 Department of Pharmacology & Cancer Biology, Duke University Medical Center, Durham, North Carolina, United States of America; 2 Department of Radiation Oncology, Duke University Medical Center, Durham, North Carolina, United States of America; Case Western Reserve University, UNITED STATES

## Abstract

In many different human cancers, one of the *HRAS*, *NRAS*, or *KRAS* genes in the RAS family of small GTPases acquires an oncogenic mutation that renders the encoded protein constitutively GTP-bound and thereby active, which is well established to promote tumorigenesis. In addition to oncogenic mutations, accumulating evidence suggests that the wild-type isoforms may also be activated and contribute to oncogenic RAS-driven tumorigenesis. In this regard, redox-dependent reactions with cysteine 118 (C118) have been found to promote activation of wild-type HRAS and NRAS. We sought to determine if this residue is also important for the activation of wild-type KRAS and promotion of tumorigenesis. Thus, we mutated C118 to serine (C118S) in wild-type KRAS to block redox-dependent reactions at this site. We now report that this mutation reduced the level of GTP-bound KRAS and impaired RAS signaling stimulated by the growth factor EGF. With regards to tumorigenesis, we also report that oncogenic HRAS-transformed human cells in which endogenous KRAS was knocked down and replaced with KRAS^C118S^ exhibited reduced xenograft tumor growth, as did oncogenic HRAS-transformed *Kras^C118S/C118S^* murine cells in which the C118S mutation was knocked into the endogenous *Kras* gene. Taken together, these data suggest a role for redox-dependent activation of wild-type KRAS through C118 in oncogenic HRAS-driven tumorigenesis.

## Introduction

The *RAS* family of small GTPases is comprised of three genes in humans, *HRAS*, *NRAS* and *KRAS* that encode the proteins HRAS, NRAS, KRAS4A and KRAS4B. Activation of growth factor receptors as well as other receptors recruits *G*uanine nucleotide *E*xchange *F*actors (GEFs), which stimulate the exchange of GDP on RAS for GTP, rendering the protein active. In this active state, RAS recruits and activates RAF kinases, PI3 kinases (PI3K), RalGEFs, and other proteins that are well known to mediate a host of cellular phenotypes, including cell proliferation and survival. RAS is, in turn, returned to the inactive GDP-bound state through association with *G*TPase *A*ctivating *P*roteins (GAPs) [1,2].

In up to a third of all human cancers, one of the three *RAS* genes harbor a mutation, typically at G12, G13 or Q61, that leads to chronic GTP binding and, correspondingly, oncogenic activation of the protein [3]. A wealth of studies demonstrate that expression of any of the RAS proteins containing an oncogenic mutation can impart transformed and tumorigenic phenotypes to cells, and when oncogenic mutations are engineered into endogenous *Ras* genes in mice, such changes can induce tumorigenesis [4].

In addition to the oncogenic RAS protein, accumulating evidence supports both tumor-suppressive and tumor-promoting roles for the remaining wild-type RAS proteins in cancer. With regards to tumor-suppressive roles, loss of one or both of any of the wild-type *Ras* alleles increases the sensitivity of mice to the carcinogen urethane, which induces lung adenomas with an oncogenic mutation in *Kras* [5,6]. With regards to tumor-promoting roles, loss of *Hras* or *Nras* renders mice more resistant to DMBA and TPA treatment, which induces skin papillomas with an oncogenic mutation in *Hras* [5]. In cultured cells, wild-type HRAS, NRAS or KRAS have been shown to promote proliferation of oncogenic RAS-driven cancer cell lines by mediating EGF signaling [7]. Knocking down Hras also sensitizes oncogenic Kras-transformed murine cells to DNA damaging chemotherapeutics [8]. Finally, wild-type KRAS has been shown to inhibit apoptosis induced by oncogenic KRAS [9].

We previously found that oncogenic KRAS stimulates the PI3K-AKT pathway [4], and activated AKT can phosphorylate S1177 of endothelial nitric oxide synthase (eNOS), activating the enzyme to produce nitric oxide (NO) [10–12]. NO as well as other free radical oxidants have been shown to facilitate *S*-nitrosylation or *S*-glutathiolation of wild-type HRAS in a manner dependent upon the thiol residue of C118, and further, such alterations activate HRAS [13–15]. Redox-dependent reactions on C118 of wild-type HRAS or NRAS can promote oncogenic KRAS-driven tumorigenesis. In more detail, substitution of C118 for serine (C118S), a very minor modification in which the thiol residue of this cysteine is replaced with a hydroxyl group, renders HRAS completely insensitive to activation by free radical oxidants, with no measureable effect on the protein structure, GTPase activity, intrinsic and GEF-mediated guanine nucleotide dissociation rate, or the ability to bind an effector [13,15–22]. Taking advantage of this very specific separation-of-function mutation to specifically block redox-dependent reactions on C118 of RAS, we previously demonstrated that knocking down wild-type HRAS or NRAS by shRNA reduced oncogenic KRAS-driven tumorigenesis in some cell lines, and that this effect was rescued by re-expressing wild-type HRAS or NRAS, but not the C118S mutant variants [23].

While blocking redox-dependent reactions on C118 of wild-type HRAS and NRAS can inhibit oncogenic KRAS-driven tumorigenesis, it was not known if the C118S mutation had the same effect on wild-type KRAS. This cysteine is conserved between HRAS, NRAS and KRAS and resides in a rather well conserved region (80% homology between amino acids 101–140 in the three RAS proteins). As such, it stands to reason that it may similarly function in an analogous fashion in KRAS. Thus, we tested whether introducing the C118S mutation into wild-type KRAS reduced the amount of GTP-bound KRAS and inhibited oncogenic HRAS-driven tumorigenesis.

## Materials and Methods

### Plasmids

pBabePuro, pBabeNeo-SV40-T/t-Ag (encoding the early region of SV40), pBabePuro-Flag-HRAS^G12V^, pBabeBleo-p110-CAAX, pBabeBleo-eNOS^S1177D^-HA and pBabeBleo-eNOS^S1177A^-HA were previously described [23,24]. pSuperRetroGFP/Neo-*KRAS*-shRNA was designed with the targeting sequence GTTGGAGCTGGTGGCGTAG. pSuperRetroGFP/Neo-scramble shRNA was designed with the sequence GATTTGGGAATCTTATAAGTTCCCTATCAGTGATAGAGATGGTCAGCGCACTCTTGCCTTTTTA. pBabe Hygro-Flag-KRAS^C118S^,-KRAS*^C118S^ and-KRAS^G12V, C118S^ were created by introducing C118S and/or G12V mutations by site-directed mutagenesis into the previously described pBabeHygro-Flag-KRAS [23] and-KRAS* [25] vectors, which encode shRNA-resistant and N-terminal Flag epitope-tagged human *KRAS4B* cDNA, either comprised of the wild-type sequence (KRAS) or one in which a number of rare codons were converted to common codons to increase protein expression (KRAS*).

### Cell lines

HEK-TtH cells (primary human embryonic kidney cells transduced with vectors encoding the early region of SV40 and hTERT) were previously described [26]. Mouse embryonic fibroblasts (MEFs) were prepared using standard approaches [27] from embryos isolated from *Kras* ^*C118S/+*^ females bred with *Kras* ^*C118S/+*^ males. Each primary MEF line was immortalized by stably infection [24] with a retrovirus derived from pBabeNeo-SV40-T/t-Ag. Genotypes of resultant MEF cell lines were determined by PCR with the primer pair P7+P8 (*see* **S1 Table**) that distinguishes the wild-type and C118S *Kras* alleles by amplification of a 621 bp versus a 517 bp product, respectively, as previously described [28]. HEK-TtH cells or SV40-immortalized *Kras* ^*+/+*^ or *Kras* ^*C118S/C118S*^ MEFs were stably infected [24] with retroviruses derived from vectors encoding no transgene as a control or encoding the indicated transgenes or shRNAs.

### Immunoblot analysis

The indicated cells were lysed with RIPA buffer (1% NP-40, 20 mM Tris pH 8.0, 137 mM NaCl, 10% glycerol and 2mM EDTA) and protein concentrations determined by Bradford assay (Bio-Rad). Equal amount of protein lysates (50 μg) were resolved by SDS-PAGE, transferred to a PVDF membrane and immunoblotted with primary antibodies anti-Kras F234 (Santa Cruz sc-30, diluted 1:200), anti-Flag M2 (Sigma F1804, diluted 1:1000), anti-HA (Covance, diluted 1:1000), anti-β-actin (Sigma A2228, diluted 1:10000), anti-β-tubulin (Sigma T5201, diluted 1:2000), anti-Erk1/2 (Santa Cruz sc-94, diluted 1:2000), anti-P(Thr 202/Tyr 204)-ERK1/2 (Santa Cruz sc-7383, diluted 1:500), anti-AKT (Cell Signaling, diluted 1:1000) or anti-P(Thr 308)-AKT (Cell Signaling, diluted 1:200), followed by incubation with either goat anti-rabbit (Santa Cruz sc-2004, diluted 1:5000) or anti-mouse (Invitrogen G21040, diluted 1:10000) IgG-HRP (Horseradish peroxidase) conjugated secondary antibodies and detected by ECL (GE healthcare). Bands of immunoblot were quantified using Image J software. Full length blots are shown in **S1 Fig.**

### Ras-GTP analysis

Cell lysates were prepared and protein concentrations determined as above from the indicated cells cultured over night in medium supplemented with 0.5% fetal bovine serum. Equal amount (0.5–2 mg) of lysates were incubated with recombinant glutathione *S*-transferase protein fused with the Ras-binding domain of Raf (GST-RBD) bound to glutathione agarose beads (GE healthcare) and rotated for 45 minutes at 4°C as previously described [29]. The beads were washed with 0.5 ml of RIPA buffer three times for 10 minutes each at 4°C, boiled in sample buffer, resolved using SDS-PAGE and immunoblotted with anti-Kras or anti-Flag antibodies, as described above. Full length blots are shown in **S1 Fig.**

### RT-PCR analysis

RNA was extracted from cells with the RNA-BEE reagent according to the manufacture’s protocol (Fisher Scientific). 0.5–2.0 μg of RNA was reverse transcribed using Omniscript RT kit (QIAGEN) with an Oligo dT (QIAGEN) primer. Resultant cDNA was used as a template to amplify targets of interest using the primers (P1-P6) listed in **S1 Table**. Full length gels are shown in **S1 Fig.**

### Tumor xenograft analysis

All animal experiments were approved by an Institutional Animal Care and Use Committee at Duke University. 5x10^6^ of the indicated HEK-TtH-derived cell lines or 1x10^6^ of the indicated SV40-immortalized MEF-derived cell lines were mixed with 500 μl Matrigel (BD Biosiences) and injected subcutaneously into each flank of five, 8-week-old female immunocompromised SCID-Bg mice (Charles River). Seven days later, the length (L) and width (W) of tumors were measured with a caliper three times a week. Tumor volumes were calculated by 0.5*L* W^2^. When the average tumor volume in one mouse in a group reached 1.5 cm^3^, all mice were euthanized and the tumors removed, photographed and weighed. For survival studies, mice were injected and monitored as above, except that each individual mouse was euthanized when tumors reached 1.5 cm^3^ or if a moribundity endpoint was reached, and time to reach endpoint was recorded. Moribundity was defined by changes in the hair coat, changes in the activity level lasting more than one week, changes in posture of ambulation lasting more than one week, changes in facial expression or weight loss >15%. Euthanasia was performed by carbon dioxide asphyxiation followed by thoractomy in accordance with the recommendations of the Panel on Euthanasia of the American Veterinary Medical Association. Kaplan-Meier survival curves were generated with Graphpad Prism 5 software.

### Soft agar growth analysis

The indicated SV40-immortalized *Kras* ^*+/+*^ and *Kras* ^*C118S/C118S*^ MEFs stably infected with a retrovirus encoding no transgene (vector) or HAS^G12V^ were plated in triplicates in 6-well plates in soft agar, as previously described [30]. Four weeks later each well was imaged, which was used to quantitate the number of colonies using Image J software.

### Statistical analysis

Data were presented as mean values ± standard error of the mean (SEM). Statistical analyses were performed with Graphpad Prism 5 software. The two-tailed unpaired student’s *t* test was used to compare two groups. The one-way ANOVA plus post-hoc Bonferroni’s multiple comparison tests were used to compare three or more groups. The long-rank test was performed to compare the survival between two groups. *P* values < 0.05 were considered significant.

## Results

### A C118S mutation introduced into wild-type KRAS impairs oncogenic HRAS^G12V^-driven tumorigenesis

**Fig 1:**
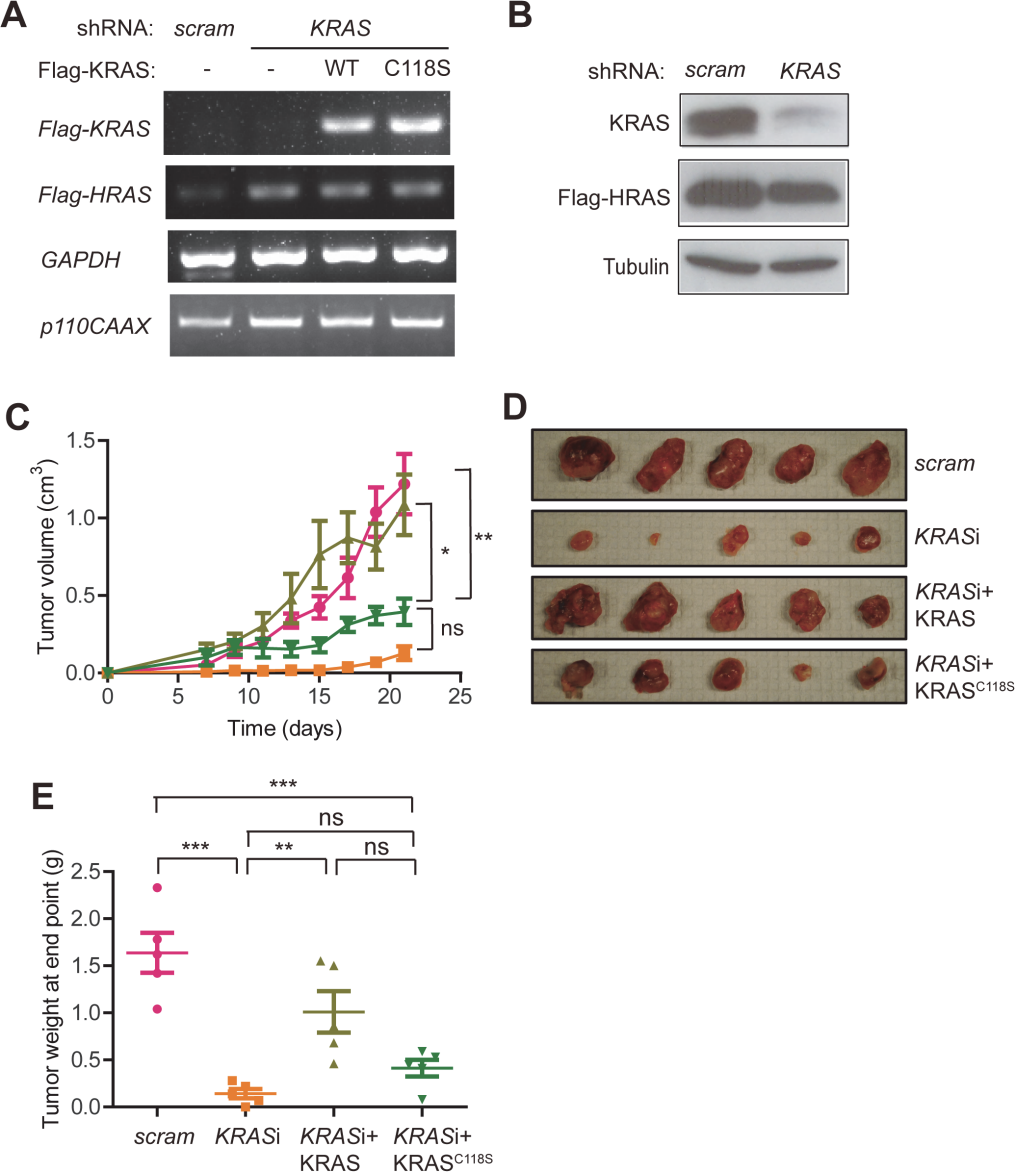
Introducing a C118S mutation into wild-type KRAS impairs HRAS^G12V^-driven tumor growth. (**A**) RT-PCR amplification of *Flag-KRAS*, *Flag-HRAS*, *p110CAAX* and *GAPDH* mRNA isolated from oncogenic Flag-HRAS^G12V^-transformed HEK-TtH cells infected with retroviruses encoding p110-CAAX and either scramble (scram) control shRNA or *KRAS* shRNA in the absence (-) or presence of shRNA-resistant wild-type (WT) or C118S mutant Flag-tagged KRAS. One of two replicate experiments. (**B**) Immunoblot detection of endogenous KRAS, Flag-tagged HRAS^G12V^ and tubulin in oncogenic Flag-HRAS^G12V^-transformed HEK-TtH cells infected with retroviruses encoding p110-CAAX and either KRAS shRNA or a scramble control (scram) shRNA. One of three replicate experiments. (**C**) Mean ± SEM tumor volume over time, (**D**) photographs of excised tumors at end point and (**E**) mean ± SEM tumor weight at end point of tumors developing in immunocompromised mice (n = 5) injected with oncogenic Flag-HRAS^G12V^-transformed HEK-TtH cells infected with retroviruses encoding p110-CAAX and either scramble control shRNA (pink circles, scram) or *KRAS* shRNA without (orange squares, *KRASi*) or in conjunction with shRNA-resistant and Flag-tagged wild-type (light green triangles, *KRASi*+KRAS) or C118S-mutant (dark green reverse triangles, *KRASi*+KRAS^C118S^) *KRAS*. ns: non-significant, *: *P*<0.05, **: *P*<0.01 and ***: *P*<0.001, as determined by one-way ANOVA plus post-hoc Bonferroni’s multiple comparison test using GraphPad Prism 5 Software. Full-length immunoblots and gels are shown in **S1A and S1B Fig**.

To evaluate the effect of blocking redox-dependent reactions with C118 in wild-type KRAS on oncogenic HRAS^G12V^-driven tumorigenesis, we monitored tumor growth of oncogenic HRAS^G12V^-transformed cells upon replacing the endogenous wild-type KRAS protein with the C118S mutant version. To create the cell lines for this experiment, oncogenic HRAS^G12V^-transformed human HEK-TtH cells [23] were stably infected with a retrovirus encoding p110-CAAX, a constitutive active PI3K, to activate AKT and promote S1177 phosphorylation and activation of eNOS [10–12]. Appropriate expression of p110-CAAX was validated by RT-PCR (**Fig 1A**). To then parse out the contribution of wild-type KRAS protein to the tumorigenic growth of these cells, this cell line was stably infected with a retrovirus encoding a scramble or *KRAS*-specific shRNA. Appropriate knockdown of endogenous wild-type KRAS was validated by immunoblot (**Fig 1B**). Resultant cells in which the expression of wild-type KRAS was knocked down were stably infected with a retrovirus encoding N-terminal, Flag epitope-tagged wild-type or C118S-mutant *KRAS4B* (termed *KRAS* hereafter for ease of discussion) that was engineered to be resistant to the *KRAS*-specific shRNA. Re-expression of wild-type and C118S Flag-KRAS was confirmed by RT-PCR (**Fig 1A**).

To next evaluate the effect of replacing endogenous wild-type KRAS with the C118S mutant form on tumorigenesis, all four of the above cell lines were each injected into the flanks of five immunocompromised mice, after which tumor growth was monitored over time. Consistent with the finding that knocking down wild-type HRAS or NRAS can reduce tumor growth of some *KRAS* mutation-positive cancer cells [23], knocking down wild-type KRAS also reduced tumor growth over time (**Fig 1C**), resulting in a statistically significant 91% reduction in tumor weight at endpoint compared to scramble shRNA control cells (**Fig 1D and 1E**). This effect was fully reversed by re-expressing shRNA-resistant wild-type Flag-KRAS, as evident from the similar tumor growth and tumor weight at endpoint between the KRAS-knockdown cells re-expressing wild-type Flag-KRAS and the scramble shRNA control cells (**Fig 1C–1E**). However, this ability of Flag-KRAS to rescue the poor tumor growth of KRAS-knockdown cells was lost if the C118S mutation was introduced into KRAS. Specifically, there was no statistical difference between KRAS-knockdown cells re-expressing Flag-KRAS^C118S^ and KRAS-knockdown cells with regards to tumor growth or tumor weight at endpoint (**Fig 1C–1E**). Thus, wild-type KRAS promotes xenograft tumor growth of oncogenic HRAS^G12V^-transformed cells expressing constitutively active PI3K in a manner dependent upon C118.

### The C118S mutation impairs activation of wild-type KRAS

**Fig 2:**
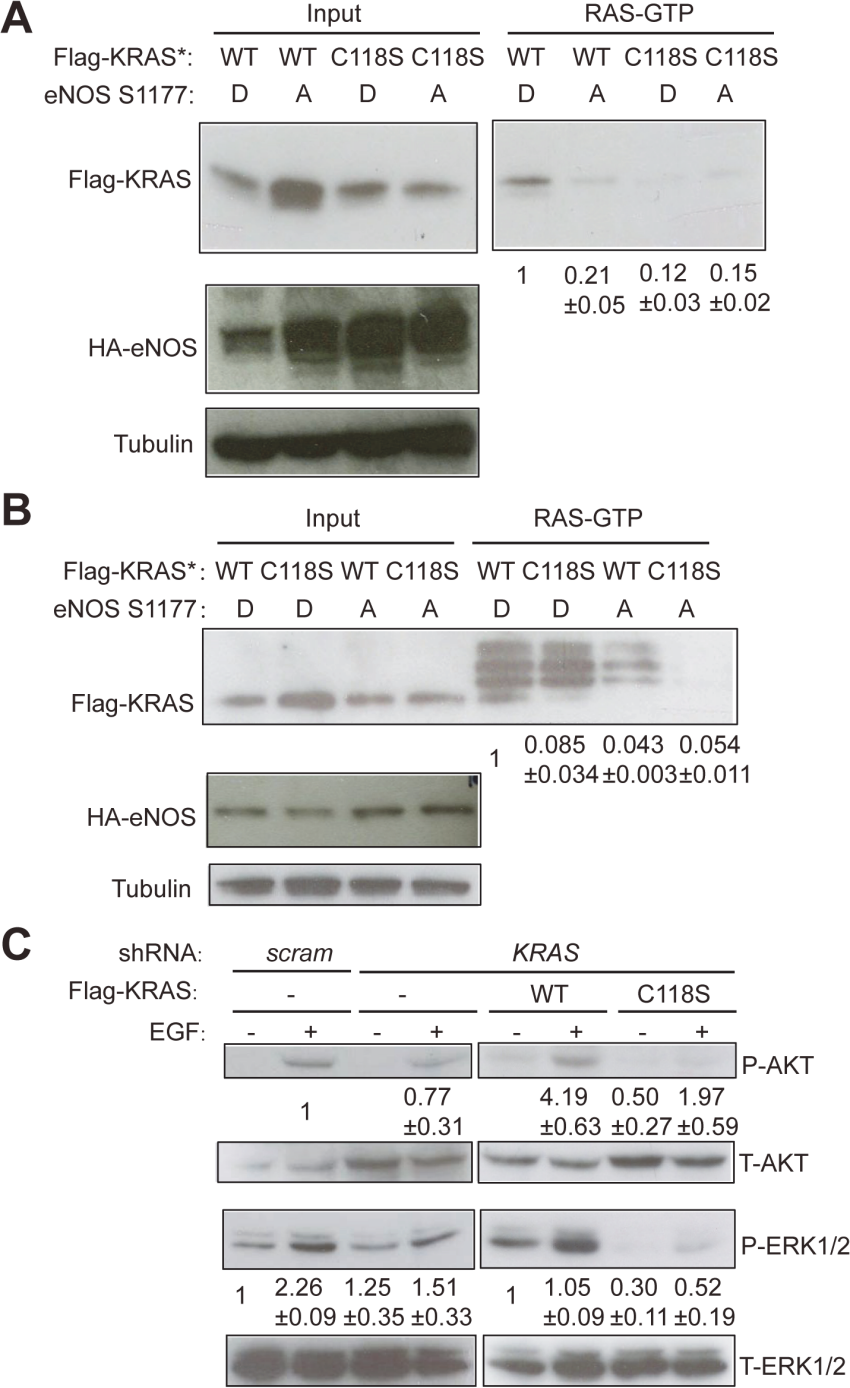
Introducing a C118S mutation into wild-type KRAS impairs EGF stimulation of AKT and ERK1/2 phosphorylation. Immunoblot detection of input and GTP-bound Flag-KRAS captured by the Ras-binding domain of Raf1, as well as HA-eNOS and tubulin, in (**A**) HEK-TtH cells or (**B**) SV40-immortalized MEFs stably infected with retroviruses encoding either wild-type (WT) or C118S-mutant Flag-tagged KRAS* in conjunction with either the HA-tagged S1177D constitutively-active or the S1177A inactive mutant versions of eNOS. High molecular weigh bands were variably detected using this assay, and because they are above the size of Ras, they were considered to be non-specific. Relative mean ± SEM of GTP-bound KRAS (normalized to KRAS input) is shown beneath the immunoblot. One of three replicate experiments. (**C**) Immunoblot detection of total (T) and phosphorylated (P) AKT and ERK1/2 in HEK-TtH cells stably infected with retroviruses encoding a scramble control (scram) shRNA, a *KRAS* shRNA, or *KRAS* shRNA and wild-type (WT) or C118S-mutant Flag-tagged shRNA-resistant KRAS and serum starved overnight and treated without (-) or with (+) EGF for five minutes. Relative mean ± SEM of P-AKT (normalized to T-AKT) or P-ERK1/2 (normalized to T-ERK) is shown beneath the immunoblot. One of three replicate experiments. Full-length immunoblots are shown in **S1C–S1E Fig.**

We previously demonstrated that the C118S mutation reduced the ability of wild-type HRAS to be activated in an eNOS-dependent fashion and to promote oncogenic KRAS-driven tumor growth [23]. As the same mutation in wild-type KRAS also reduced oncogenic HRAS^G12V^-driven tumorigenic growth (**Fig 1**), we reasoned that this may similarly be due to the C118S mutation blocking activation of wild-type KRAS. To test this possibility, we measured the amount of active (GTP-bound) KRAS in the absence or presence of the C118S mutation in cells expressing eNOS. To create the cell lines for this experiment, HEK-TtH cells were stably infected with retroviruses encoding a C-terminal, HA epitope-tagged S1177D constitutively-active [11] or S1177A inactive [11] mutant form of eNOS in conjunction with the wild-type or C118S mutant form of Flag-KRAS*, a N-terminal, Flag epitope-tagged version of KRAS optimized for expression [25]. Ectopic expression of HA-eNOS and Flag-KRAS was validated by immunoblot analysis (**Fig 2A**).

To next assess whether the C118S mutation inhibited the activation of Flag-KRAS* downstream of eNOS, GTP-bound RAS proteins were captured from all four of these cell lines using the RAS-binding domain of RAF1 [29], followed by immunoblot with an anti-Flag antibody to specifically detect ectopic Flag-KRAS*. Consistent with the findings that HRAS and NRAS are activated downstream of eNOS [23], the level of GTP-bound Flag-KRAS* was higher in cells expressing HA-eNOS^S1177D^ compared to those expressing HA-eNOS^S1177A^, even though the latter cells had higher expression of both Flag-KRAS* and HA-eNOS^S1177A^. Importantly, this activation could be ascribed to C118, as the level of GTP-bound Flag-KRAS*^C118S^ remained low, regardless of whether cells express HA-eNOS^S1177D^ or HA-eNOS^S1177A^ (**Fig 2A**).

To determine whether the effect of C118S on the level of GTP-bound KRAS was reproducible, we repeated the same experiment in another cell line. Specifically, SV40-immortalized mouse embryonic fibroblasts (MEFs) were stably infected with retroviruses encoding either Flag-KRAS* or Flag-KRAS*^C118S^ in conjunction with either HA-eNOS^S1177D^ or HA-eNOS^S1177A^. Appropriate expression of these transgenes was verified by immunoblot (**Fig 2B**). As above, cells expressing Flag-KRAS* and HA-eNOS^S1177D^ exhibited higher levels of GTP-bound ectopic Flag-KRAS than cells expressing Flag-KRAS* and HA-eNOS^S1177A^ or cells expressing Flag-KRAS*^C118S^ and HA-eNOS^S1177D^ or HA-eNOS^S1177A^ (**Fig 2B**). Thus, the C118S mutation impairs eNOS-dependent activation of wild-type KRAS in two independent types of cells.

### The C118S mutation impairs signaling by wild-type KRAS

GTP-bound RAS proteins undergo a conformational change that results in binding effector proteins like Rafs and PI3K, leading to activation of the MAPK and PI3K pathways, respectively [31,32]. Since introducing the C118S mutation into KRAS reduced the level of GTP-bound KRAS detected in cells expressing activated eNOS (**Fig 2A and 2B**), we tested whether this mutation also impeded the ability of KRAS to activate effector pathways. To this end, the aforementioned oncogenic HRAS^G12V^-transformed HEK-TtH cells expressing p110-CAAX and either scramble control or *KRAS* shRNA in the absence or presence of shRNA-resistant Flag-KRAS or Flag-KRAS^C118S^ were serum starved over night. Lysates were then collected and immunoblotted for total (T-) and phosphorylated (P-) AKT and ERK1/2, markers of activation of the PI3K and MAPK RAS effector pathways [33]. While knocking down KRAS did not have a marked effect on the basal levels of P-AKT and P-ERK1/2 compared to scramble control cells, there was a reduction in basal P-ERK1/2 levels in the cells reconstituted with Flag-KRAS^C118S^ compared to Flag-KRAS. Wild-type RAS proteins can be activated by Epidermal Growth Factor (EGF) in the presence of oncogenic RAS [7]. Thus, we also measured the levels of P-AKT and P-ERK1/2 upon EGF stimulation. In this case, knocking down endogenous wild-type KRAS did reduce the level of P-AKT and P-ERK1/2 induced by EGF compared to scramble control cells. Moreover, stimulation of AKT and ERK1/2 phosphorylation by EGF in KRAS-knockdown cells was restored by re-expressing Flag-KRAS, but not Flag-KRAS^C118S^ (**Fig 2C**). We also note here that the level of P-AKT and P-ERK1/2 was reduced more by expressing KRAS^C118S^ than by knocking down endogenous wild-type KRAS, which is similar to reports by others [20,34–36], suggestive of a possible dominant-negative effect by ectopic KRAS^C118S^. Thus, the C118S mutation impairs signaling by wild-type KRAS in HRAS^G12V^-transformed cells, primarily in the setting of EGF stimulation.

### An activating mutation in wild-type KRAS overcomes the reduction of tumor growth imparted by the C118S mutation

**Fig 3:**
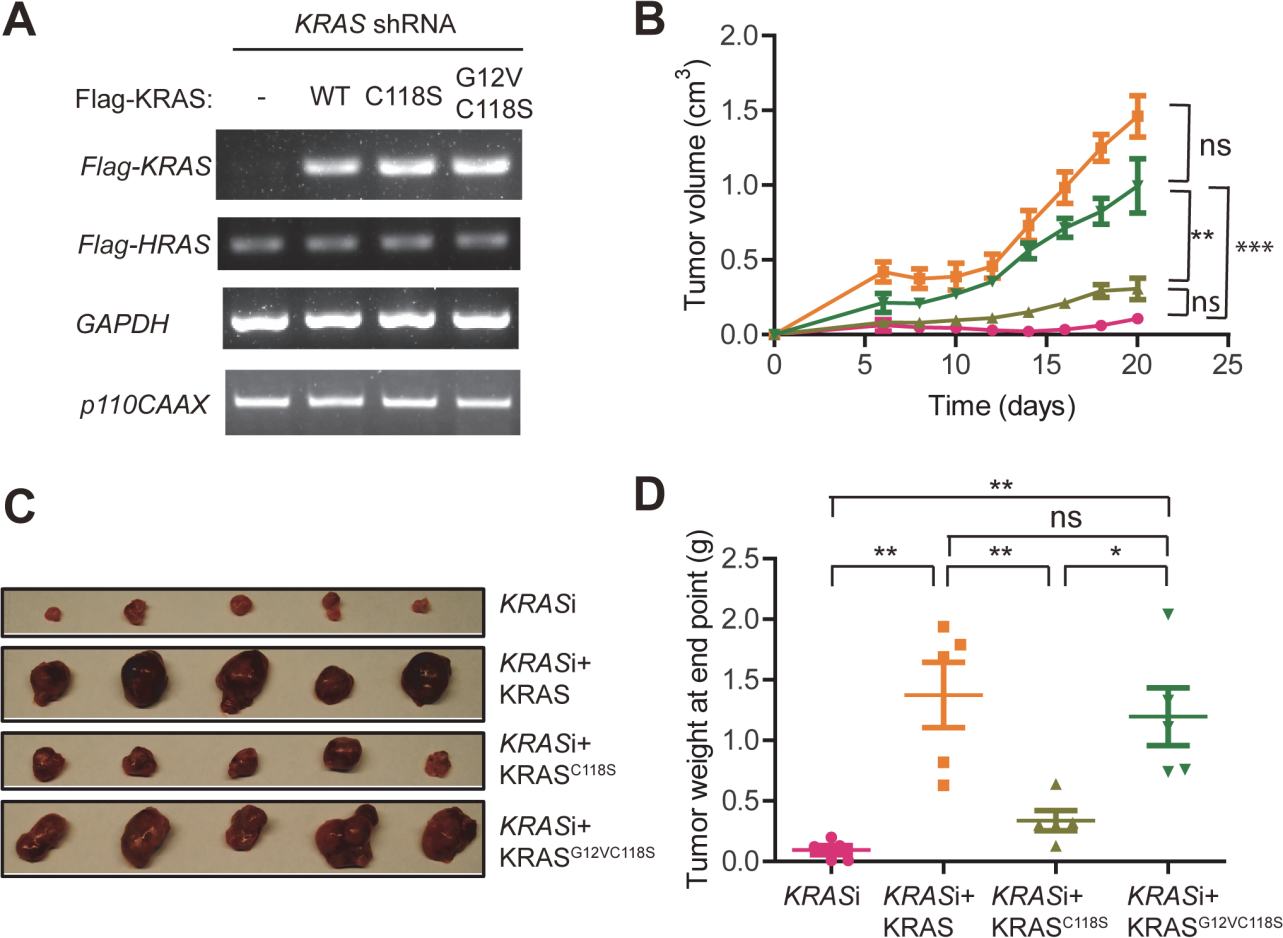
Introducing a G12V activating mutation overcomes the inability of KRAS^C118S^ to promote HRAS^G12V^-driven tumor growth. (**A**) RT-PCR amplification of *Flag-KRAS*, *Flag-HRAS*, *p110CAAX* and *GAPDH* mRNA (note that this is the same gel as in **Fig 1A**, except with the addition of analysis for Flag-KRAS^G12V,C118S^, one of two replicate experiments), (**B**) mean ± SEM tumor volume over time, (**C**) photographs of excised tumors at end point and (**D**) mean ± SEM tumor weight at end point of tumors developing in immunocompromised mice (n = 5) injected with Flag-HRAS^G12V^-transformed HEK-TtH cells infected with retroviruses encoding p110CAAX and *KRAS* shRNA alone (pink circles) or with wild-type (WT, orange squares), C118S-mutant (light green triangles) or G12V,C118S-mutant (dark green reverse triangles) Flag-tagged and shRNA-resistant KRAS. ns: non-significant, *: *P*<0.05, **: *P*<0.01 and ***: *P*<0.001, as determined by one-way ANOVA plus post-hoc Bonferroni’s multiple comparison test using GraphPad Prism 5 Software. The full-length gel is shown in **S1A Fig.**

The findings that the C118S mutation inhibited the activation and signaling as well as tumorigenesis mediated by wild-type KRAS argues that these effects are related. To genetically test whether the defect inflicted by the C118S mutation could be overcome by activating KRAS^C118S^, the above oncogenic HRAS^G12V^-transformed HEK-TtH cells expressing p110-CAAX and KRAS shRNA were engineered to express no other transgene or shRNA-resistant Flag-tagged KRAS, KRAS^C118S^ or KRAS^C118S^ with an activating (G12V) mutation. Re-expression of the three versions of Flag-KRAS was confirmed by RT-PCR (**Fig 3A**). The four resultant cell lines were then each injected into the flanks of five immunocompromised mice, after which tumor growth was monitored over time. As already observed (**Fig 1**), the reduced tumor growth of the KRAS-knockdown cells was reversed by re-expressing shRNA-resistant wild-type Flag-KRAS, as these cells formed significantly larger (7.3 fold) tumors than the KRAS-knockdown cells. Again, re-expressing the C118S mutant version did not rescue this phenotype, as evidenced by similar tumor growth and tumor size and weight at endpoint in tumors derived from the KRAS-knockdown cells and the KRAS-knockdown cells expressing Flag-KRAS^C118S^ (**Fig 3B–3D**). Importantly, the inability of Flag-KRAS^C118S^ to restore the reduced tumor growth upon knocking down endogenous wild-type KRAS was rescued if Flag-KRAS^C118S^ was engineered to contain an activating (G12V) mutation, as evident from the similar growth kinetics (**Fig 3B**) and tumor size and weight at endpoint between the KRAS-knockdown cells expressing wild-type Flag-KRAS and those expressing Flag-KRAS^C118S,G12V^ (**Fig 3B–3D**). These results support the contention that the C118S mutation blocks activation of wild-type KRAS and the ability of this protein to support tumor growth.

### The C118S mutation introduced into the endogenous murine wild-type *Kras* gene impairs oncogenic HRAS^G12V^-driven transformation

**Fig 4:**
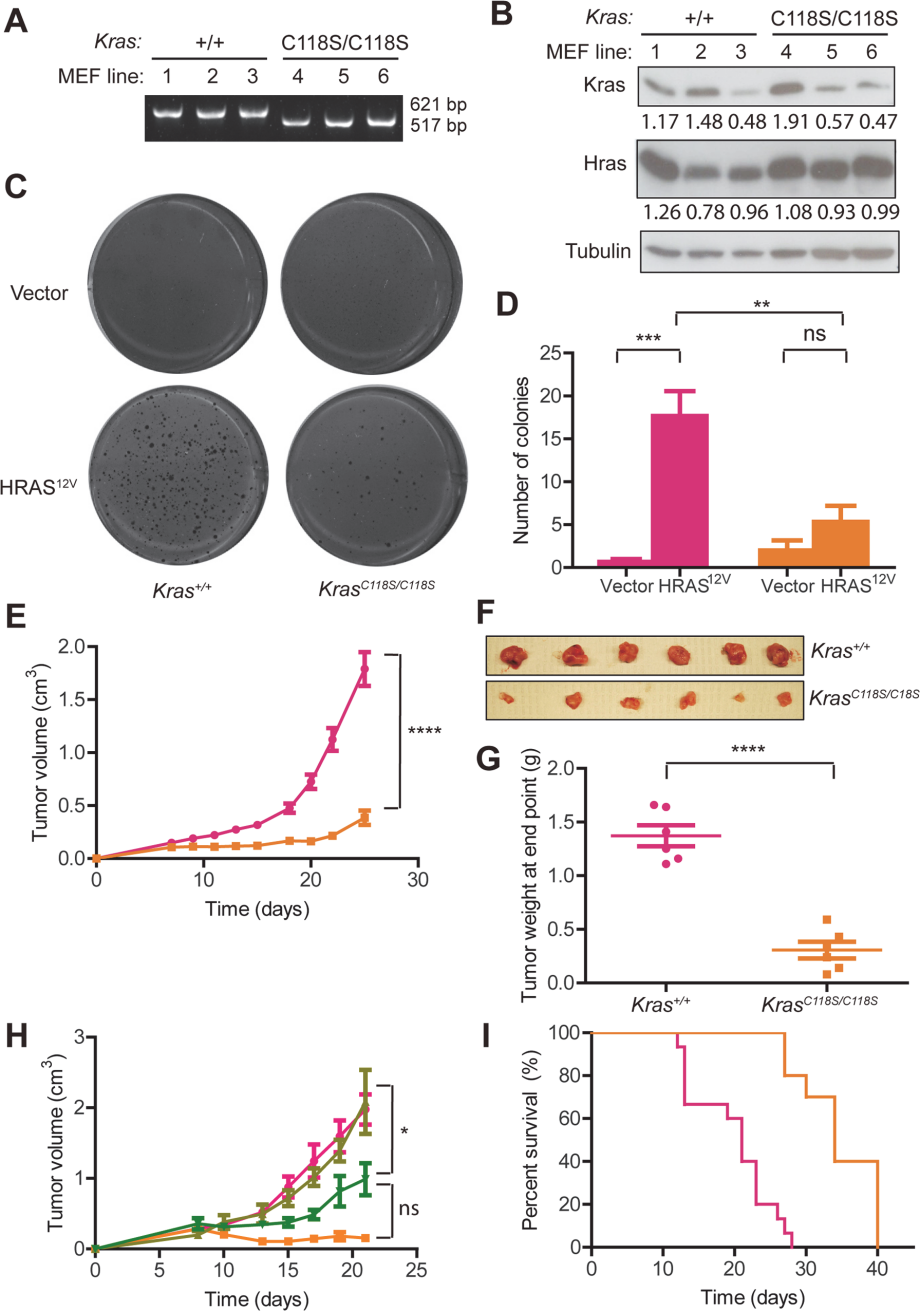
Introducing a C118S mutation into the endogenous wild-type *Kras* gene impairs HRAS^G12V^-driven tumor growth. (**A**) PCR amplification of genomic DNA yielding a 621 bp or 517bp fragment indicative of the wild-type or C118S *Kras* alleles in three SV40-immortalized *Kras* ^*+/+*^ or *Kras* ^*C118S/C118S*^ MEF cell lines, respectively. (**B**) Immunoblot detection of endogenous Kras, HRAS and tubulin in the indicated SV40-immortalized *Kras* ^*+/+*^ or *Kras* ^*C118S/C118S*^ MEF cell lines transformed with HRAS^G12V^. Relative Kras or HRAS levels normalized to tubulin are shown beneath the immunoblot. (**C**) Representative images and (**D**) the mean ± SEM number of colonies growing in soft agar by SV40-immortalized *Kras* ^*+/+*^ versus *Kras* ^*C118S/C118S*^ MEF cell lines stably infected with a retrovirus encoding no transgene (vector) or HRAS^G12V^, seeded in triplicate. (**E**) Mean ± SEM tumor volume over time, (**F**) photographs of excised tumors at end point and (**G**) mean ± SEM tumor weight at end point of tumors developing in immunocompromised mice (n = 5) injected with SV40-immortalized *Kras* ^*+/+*^ (pink boxes) versus *Kras* ^*C118S/C118S*^ (orange squares) MEF cell lines transformed with HRAS^G12V^. (**H**) Mean ± SEM tumor volume over time of tumors developing in immunocompromised mice (n = 5) injected with SV40-immortalized and HRAS^G12V^-transformed *Kras* ^*+/+*^ MEFs (pink circles), *Kras* ^*C118S/C118S*^ MEFs (orange squares) or *Kras* ^*C118S/C118S*^ MEFs stably infected with a retrovirus encoding KRAS* (light green triangles) or KRAS*^C118S^ (dark green reverse triangles). (**I**) Kaplan-Meier survival curves based on the time to reach end point of immunocompromised mice (n = 5) each injected with one of the three SV40-immortalized *Kras* ^*+/+*^ (pink line) versus *Kras* ^*C118S/C118S*^ (orange line) MEF cell lines transformed with HRAS^G12V^. ns: non-significant, **: *P*<0.01, ***: *P*<0.001 and ****: *P*<0.0001, as determined by one-way ANOVA plus post-hoc Bonferroni’s multiple comparison test (D, H), two-tailed unpaired *t* test (E, G) or long-rank test (I) using GraphPad Prism 5 Software. Full-length immunoblots and gels are shown in **S1F and S1G Fig.**

Admittedly, one caveat to the above experiments was that KRAS was restored in KRAS-knockdown cells by ectopic expression of various versions of KRAS protein. Thus, to assess the effect on tumorigenesis when the C118S mutation was introduced into the endogenous *Kras* gene, MEFs were isolated from three *Kras* ^*C118S/C118S*^ mouse embryos in which the C118S mutation was knocked into both alleles of the endogenous *Kras* gene [28], as well as three control *Kras* ^*+/+*^ embryos. These six cultures were immortalized by stable infection with a retrovirus encoding the early region of SV40, and the *Kras* genotype confirmed by PCR in the resultant immortalized MEF cell lines (**Fig 4A**). Immunoblot analysis revealed varying levels of Kras protein regardless of the genotype (**Fig 4B**). Given this, *Kras* ^*+/+*^ cell line 3 and *Kras* ^*C118S/C118S*^ cell line 5 were chosen for analysis, owing to their similar levels of Kras protein expression. These two cell lines were stably infected with a retrovirus encoding no transgene (vector) or oncogenic HRAS^G12V^. Appropriate expression of oncogenic HRAS^G12V^ was validated by immunoblot (**Fig 4B**). The four resultant cell lines were then assayed in triplicate for the transformed phenotype of growth in soft agar. As expected, vector control cells did not grow in soft agar, but introduction of oncogenic HRAS^G12V^ permitted this growth. However, there was a statistically significant, 70% reduction in the mean number of colonies seeded by oncogenic HRAS^G12V^-transformed *Kras* ^*C118S/C118S*^ MEFs compared to *Kras* ^*+/+*^ MEFs (**Fig 4C and 4D**). Thus, introduction of the C118S mutation into the endogenous *Kras* gene inhibits oncogenic HRAS^G12V^-mediated transformation.

### The C118S mutation introduced into the endogenous murine wild-type *Kras* gene impairs oncogenic HRAS^G12V^-driven tumorigenesis

To assess whether the reduction in growth in soft agar observed with the oncogenic HRAS^G12V^-transformed *Kras* ^*C118S/C118S*^ MEFs reflected a defect in the more relevant *in vivo* phenotype of tumor growth, these cells and the control oncogenic HRAS^G12V^-transformed *Kras* ^*+/+*^ MEFs were injected into the flanks of five immuno-compromised mice each, after which tumor growth was monitored over time. This analysis revealed that tumors derived from oncogenic HRAS^G12V^-transformed *Kras* ^*C118S/C118S*^ MEFs grew more slowly than the control *Kras* ^*+/+*^counterparts (**Fig 4E**), which was manifested at endpoint as smaller tumors (**Fig 4F**) that weighed 78% significantly less (**Fig 4G**). As a control, we demonstrate that re-expression of ectopic wild-type KRAS* was more effective than KRAS*^C118S^ at restoring tumor growth of oncogenic HRAS^G12V^-transformed *Kras* ^*C118S/C118S*^ MEFs (**Fig 4H**). Finally, to assess the impact of the C118S mutation on the clinically relevant endpoint of survival, the above three *Kras* ^*+/+*^ and three *Kras* ^*C118S/C118S*^ immortalized MEFs were also transformed with oncogenic HRAS^G12V^ and the resultant six cell lines were each injected into the flank of five immunocompromised mice. Mice were then euthanized if they reached a maximum tumor volume or moribundity. A plot of the percent of mice surviving over time revealed that mice injected with oncogenic HRAS^G12V^-transformed *Kras* ^*C118S/C118S*^ MEFs exhibited a 62% greater, significant survival advantage over mice injected with the oncogenic HRAS^G12V^-transformed *Kras* ^*+/+*^ MEFs (**Fig 4I**). Thus, introducing the C118S mutation into the endogenous *Kras* gene inhibits oncogenic HRAS^G12V^-mediated xenograft tumor growth.

## Discussion

We report here that xenograft tumor growth of an oncogenic HRAS^G12V^-transformed human cell line engineered to express activated PI3K is diminished upon knockdown of endogenous wild-type KRAS, and that this effect was rescued by re-expressing the wild-type, but not the C118S-mutant KRAS. Oncogenic HRAS^G12V^-transformed *Kras* ^*C118S/C118S*^ MEFs also exhibited reduced anchorage-independent and tumorigenic growth compared to similarly transformed *Kras* ^*+/+*^ MEFs. Again, there was a trend towards these cells being more tumorigenic upon re-expression wild-type KRAS compared to C118S-mutant KRAS. Thus, introducing the C118S mutation into wild-type KRAS inhibits oncogenic HRAS^G12V^-mediated tumorigenesis in two different experimental settings. The same C118S mutation engineered into HRAS and NRAS was previously shown to reduce the tumor growth of the *KRAS* mutation-positive human pancreatic cancer cell line CFPac-1 [23]. As such, mutating C118 in the different wild-type RAS proteins may reduce tumorigenesis driven by different oncogenic RAS isoforms.

We also demonstrate that the level of GTP-bound wild-type KRAS in the presence of mutationally-active eNOS was reduced when the C118S mutation was introduced into the *KRAS* transgene. Moreover, EGF stimulation of the MAPK and PI3K pathways in HRAS^G12V^-transformed human cells was blunted when endogenous KRAS was replaced with the C118S mutant version. It was previously demonstrated that knocking down endogenous eNOS reduced the level of *S*-nitrosylated and GTP-bound HRAS and NRAS in cells expressing p110-CAAX to activate eNOS [23]. There is also accumulating evidence that wild-type RAS proteins are activated in cells harboring an oncogenic RAS mutation [7,23,37]. In this regard, we genetically demonstrate that the inability of KRAS^C118S^ to restore tumor growth of HRAS^G12V^-transformed human cells upon knocking down endogenous KRAS could be restored, in large part, by introducing an activating mutation into the *KRAS* ^*C118S*^ transgene. As the C118S mutation does not alter other known activities of RAS aside from activation by redox-dependent reactions [13,15–22], these results collectively support the contention that activation of eNOS through the PI3K/AKT signaling arm of oncogenic RAS leads to *S*-nitrosylation or other redox-dependent reactions with C118 in the remaining wild-type RAS proteins, leading to their activation and promotion of tumorigenesis.

The effect of the C118S mutation on the ability of wild-type KRAS to promote HRAS oncogenesis may nevertheless be dependent upon the stage of tumorigenesis or types of cancer. Specifically, we previously demonstrated that *Kras* ^*C118S/C118S*^ mice are more resistant to urethane-induced lung tumorigenesis that their *Kras* ^*+/+*^ counterparts, this effect was linked to the oncogenic, rather than the remaining non-mutated *Kras* ^*C118S*^ allele [28]. These results suggest that either later stages of tumorigenesis, such as those modeled in cancer cell lines, or specific types of cancer are sensitive to loss of redox-dependent reactions with C118 of wild-type KRAS. In agreement, knocking out wild-type *Ras* genes in mice can be either tumor promoting or suppressing depending upon the carcinogen, cancer type or the *Ras* isoform acquiring the oncogenic mutation [5]. Nevertheless, in the settings studied, we propose that redox-dependent reactions with C118 of wild-type KRAS activate the protein to stimulate xenograft tumor growth of oncogenic HRAS^G12V^-driven cell lines. These results, coupled with previous findings of a similar role for wild-type HRAS and NRAS in oncogenic KRAS-driven tumorigenesis [8,23,37], suggest that activation of wild-type RAS isoforms can promote oncogenic RAS-driven tumorigenesis in certain settings.

## Supporting Information

S1 Fig Full-length gels and blots for figures. Full-length gels or blots for (**A**) Fig 1A and Fig 3A, (**B**) Fig 1B, (**C**) Fig 2A, (**D**) Fig 2B, (**E**) Fig 2C, (**F**) Fig 4A, and (**G**) Fig 4B.
(PDF)

S1 Table PCR primers. (DOCX)
