# Supplementary material for: Reduced HRASG12V-Driven Tumorigenesis of Cell Lines Expressing KRASC118S
Source: PLoS One. 2015 Apr 22;10(4):e0123918. doi: 10.1371/journal.pone.0123918 (PMC4406447; doi:10.1371/journal.pone.0123918)
Supplement: S1 Fig — Full-length gels or blots for (A) Fig 1A and Fig 3A, (B) Fig 1B, (C) Fig 2A, (D) Fig 2B, (E) Fig 2C, (F) Fig 4A, and (G) Fig 4B. (PDF) [file pone.0123918.s001.pdf]

A Fig. 1A and Fig. 3A

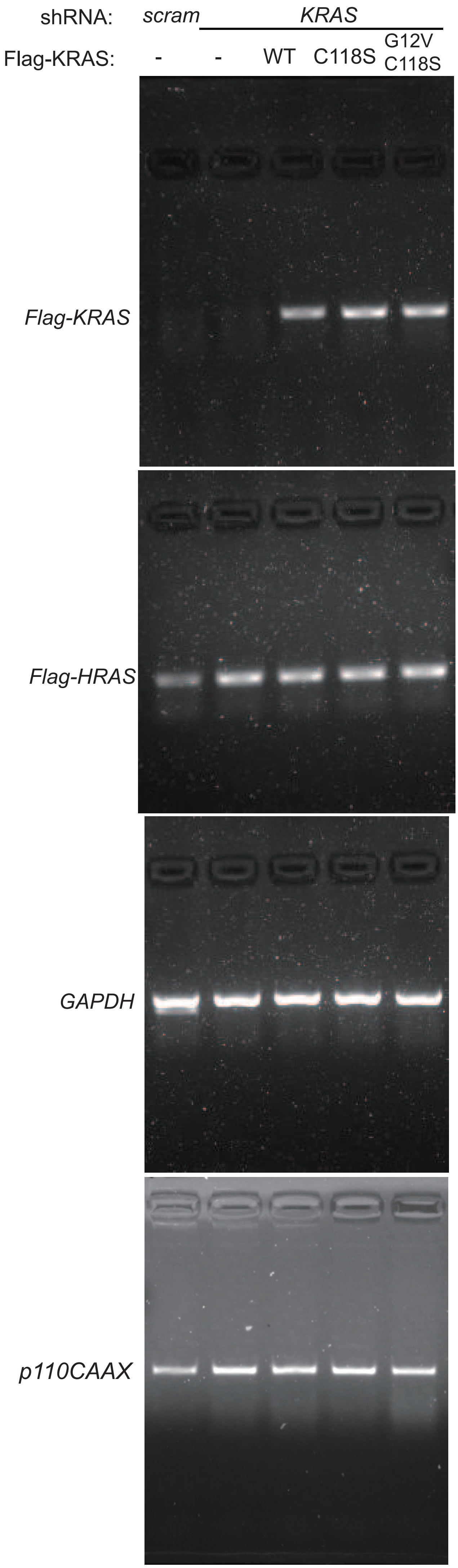

B Fig. 1B

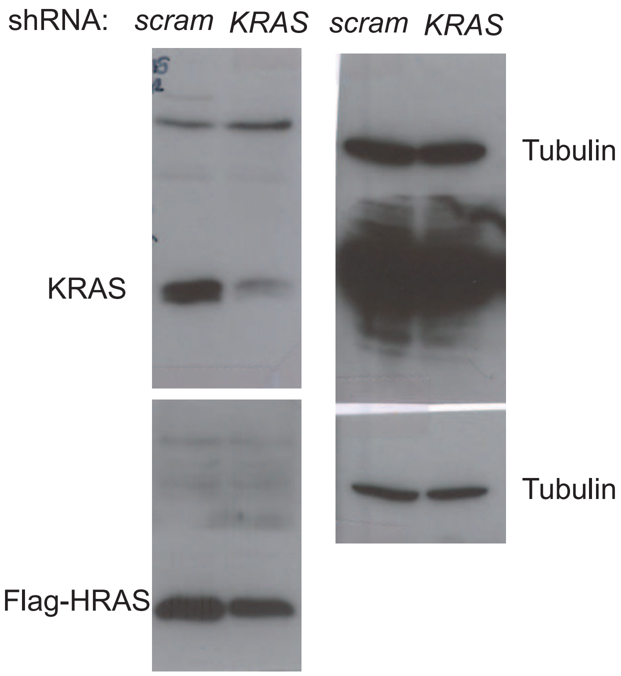

C

Fig. 2A

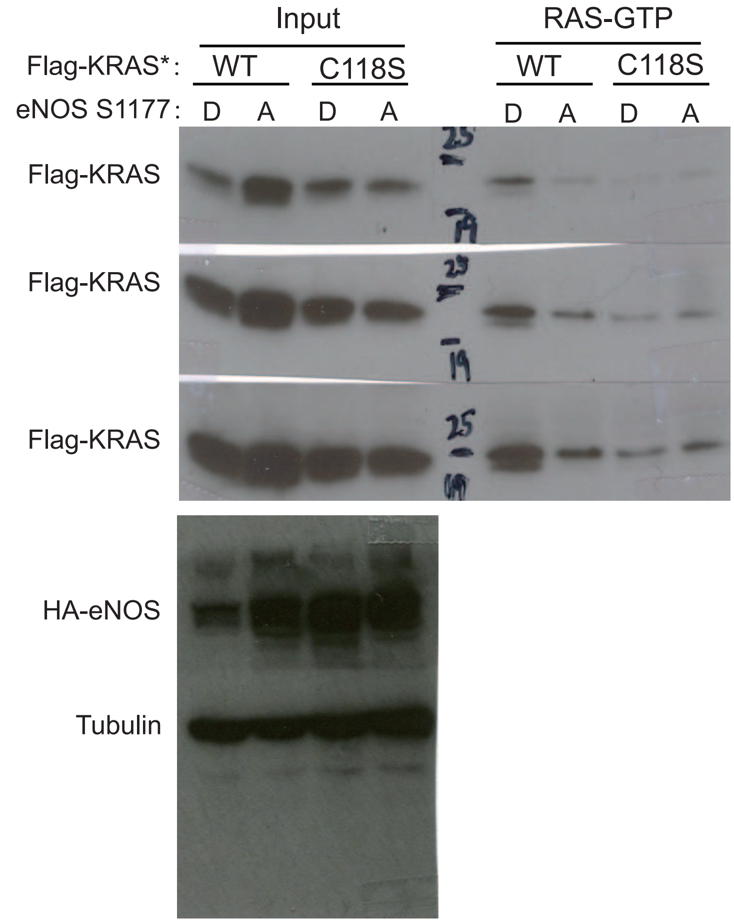

D

Fig. 2B

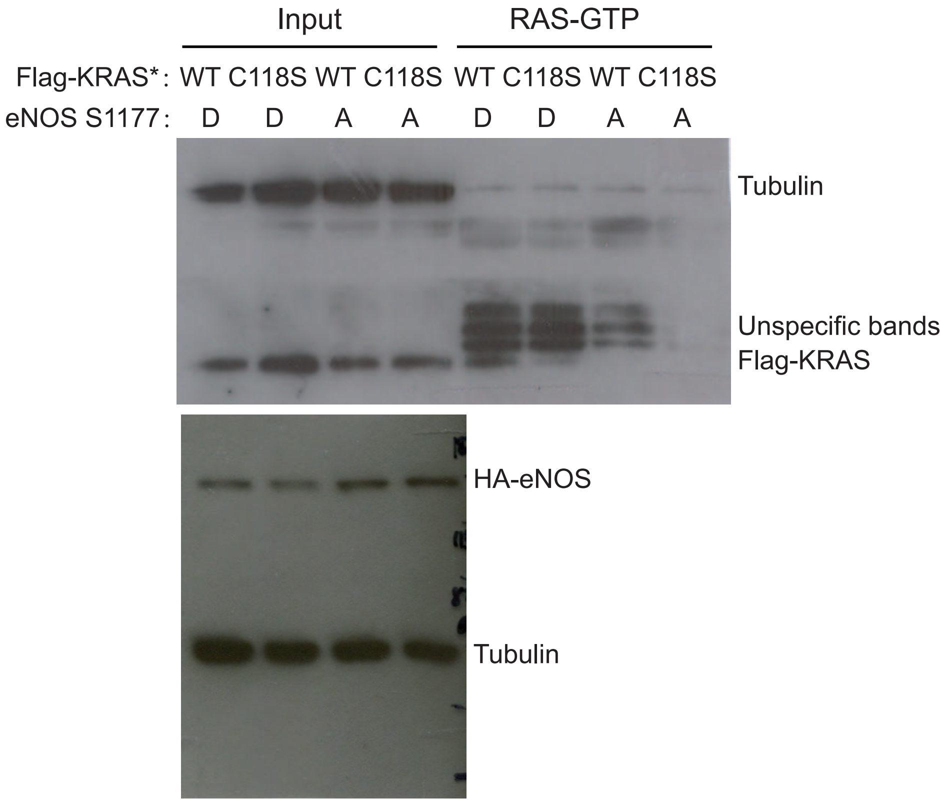

E

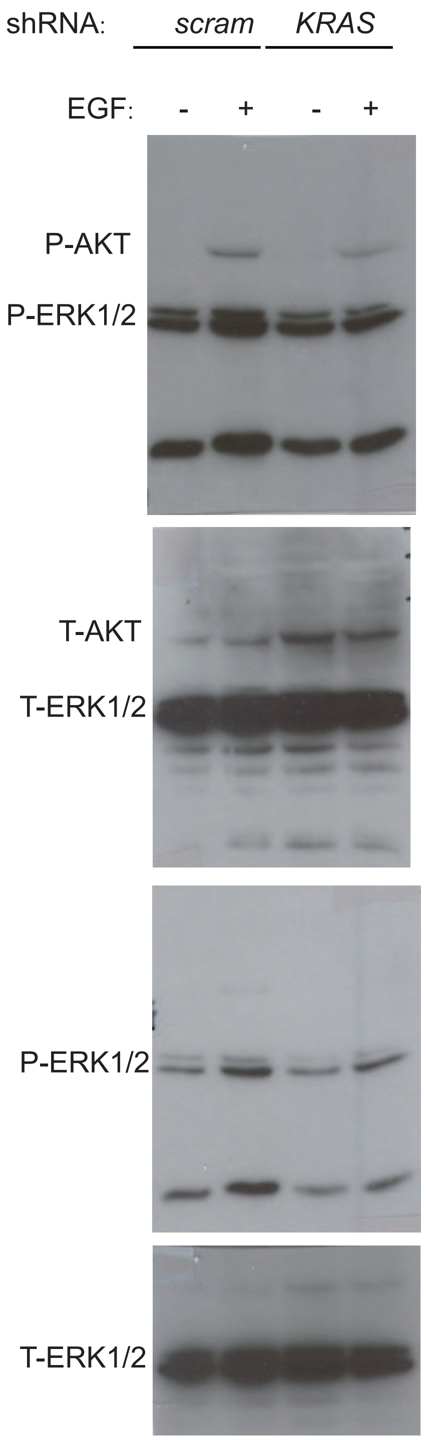
shRNA: scram KRAS
EGF: - + - +
P-AKT
P-ERK1/2
T-AKT
T-ERK1/2
P-ERK1/2
T-ERK1/2

Fig. 2C

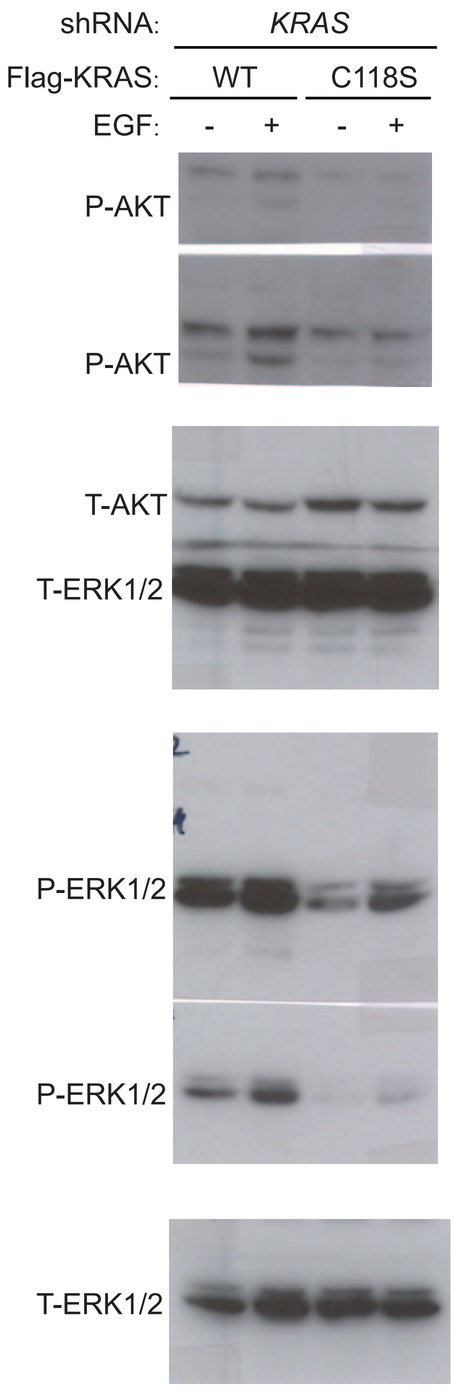
shRNA: KRAS
Flag-KRAS: WT C118S
EGF: - + - +
P-AKT
P-AKT
T-AKT
T-ERK1/2
P-ERK1/2
P-ERK1/2
T-ERK1/2

F

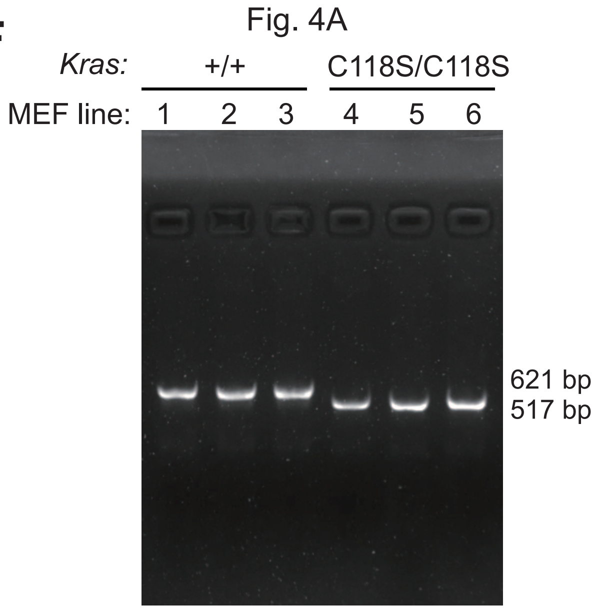

G

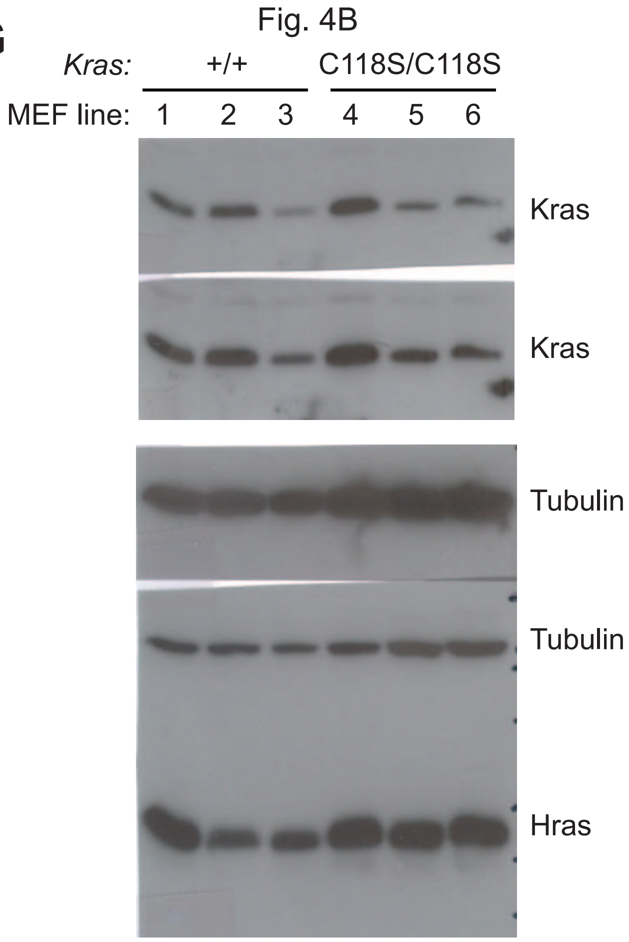

S1 Fig. Full-length gels and blots for figures.
Full-length gels or blots for (**A**) Fig. 1A and Fig. 3A, (**B**) Fig. 1B, (**C**) Fig. 2A, (**D**) Fig. 2B, (**E**) Fig. 2C, (**F**) Fig. 4A, and (**G**) Fig. 4B.
