# Supplementary material for: Reduced HRASG12V-Driven Tumorigenesis of Cell Lines Expressing KRASC118S
Source: PLoS One. 2015 Apr 22;10(4):e0123918. doi: 10.1371/journal.pone.0123918 (PMC4406447; doi:10.1371/journal.pone.0123918)
Supplement: S1 Table — (DOCX) [file pone.0123918.s002.docx]

**S1 Table. PCR primers.**

| Primer | Sequence | |
| --- | --- | --- |
| P1 (*Flag-Kras* RT-PCR F) | | aaagatgacgacgataagactgaa |
| P2 (*Flag-Kras* RT-PCR R) | | gctgtgtcgagaatatccaagaga |
| P3 (*Flag-Hras* RT-PCR F) | | agatgacgacgataagacggaata |
| P4 (*Flag-Hras* RT-PCR R) | | ggaatcctctatagtggggtcgta |
| P5 (*GAPDH* RT-PCR F) | | gaaggtgaaggtcggagtcaa |
| P6 (*GAPDH* RT-PCR R) | | gcagagggggcagagatgat |
| P7 (*Kras^C118S^* genotyping F) | | agaacaaattaaaagagtaaaggac |
| P8 (*Kras^C118S^* genotyping R-*Kras* genomic) | | atgtaaaatgtactctagacggaac |
